# Thermal-Infrared Pedestrian ROI Extraction through Thermal and Motion Information Fusion

**DOI:** 10.3390/s140406666

**Published:** 2014-04-10

**Authors:** Antonio Fernández-Caballero, María T. López, Juan Serrano-Cuerda

**Affiliations:** 1 Departamento de Sistemas Informáticos, Universidad de Castilla-La Mancha, 02071-Albacete, Spain; E-Mail: Maria.LBonal@uclm.es; 2 Instituto de Investigación en Informática de Albacete, 02071-Albacete, Spain; E-Mail: jserranocuerda@gmail.com

**Keywords:** thermal-infrared video, pedestrians, segmentation, ROI extraction, evaluation

## Abstract

This paper investigates the robustness of a new thermal-infrared pedestrian detection system under different outdoor environmental conditions. In first place the algorithm for pedestrian ROI extraction in thermal-infrared video based on both thermal and motion information is introduced. Then, the evaluation of the proposal is detailed after describing the complete thermal and motion information fusion. In this sense, the environment chosen for evaluation is described, and the twelve test sequences are specified. For each of the sequences captured from a forward-looking infrared FLIR A-320 camera, the paper explains the weather and light conditions under which it was captured. The results allow us to draw firm conclusions about the conditions under which it can be affirmed that it is efficient to use our thermal-infrared proposal to robustly extract human ROIs.

## 1. Introduction

The detection of pedestrians is a key application in the video surveillance domain [1]. Indeed, a number of surveillance applications require the detection and tracking of people to ensure security and safety [2,3]. The most widespread sensor technology for detecting pedestrians is for sure the use of gray scale [4,5] and color cameras [6,7]. However, using the visible-light information is problematic when facing quick changes in lighting or illumination problems. Now, thermal-infrared images have a number of distinctive features compared to frames acquired by a visible-light spectrum camera [8–11].

In thermal-infrared video, the gray level value of the objects is set by their temperature and radiated heat, and is independent from lighting conditions. The most intuitive idea when performing a pedestrian detection algorithm in the thermal-infrared spectrum is to take advantage of the fact that humans usually appear warmer than other objects in the scene [12,13]. However, this is not always the case [14]. The main reason is that the properties of the objects in the scene (*i.e.*, emissivity, reflectivity and transmissivity) and their wavelength affect the infrared images' intensity, especially in summer afternoon. Obviously, the condition is usually well satisfied during winter and at night. These drawbacks make it impossible to detect humans exclusively using their intensity value. On the other hand, a great amount of infrared images have low spatial resolution and lower sensitivity than visible spectrum images due to the technological limitations of thermal-infrared cameras. These defects often result in low image quality and a great amount of image noise.

Many approaches in this spectrum combine appearance and shape properties since humans are initially detected according to the former (their appearance is usually brighter than other objects in the scene) and are filtered and classified based on the latter [15]. This paper introduces a new algorithm for robust ROI extraction of pedestrians in thermal-infrared video based on the authors' previous works [16,17]. In addition to presenting the algorithm, the main objective of this article is to draw firm conclusions about the environmental conditions under which it can be affirmed that it is efficient to use thermal-infrared cameras to robustly detect pedestrians.

The rest of the article is organized as follows: Section 2 describes the new algorithm for pedestrian ROI extraction in the thermal-infrared spectrum. In Section 3 the algorithm is applied to twelve different video sequences recorded under very different environmental conditions. This way it is possible to determine which the suited ambient conditions are for using a thermal-infrared sensor in the proposed monitoring task. Finally, some conclusions are provided in Section 4.

## 2. Pedestrian ROI Extraction in Thermal-Infrared Video

As previously explained, the infrared spectrum has many interesting features which can be exploited for robust human detection. Two of these properties are clearly important: (1) the independence of lighting conditions of the scene, and specially, (2) the fact that humans tend to be clearly highlighted respect to the background of the picture. Usually, humans' heads also appear hotter than the rest of the body covered with clothes. This is why a *Thermal Analysis* is developed using these properties on each single frame of the video, that is, the current image frame, *I*(t).

**Figure 1.**
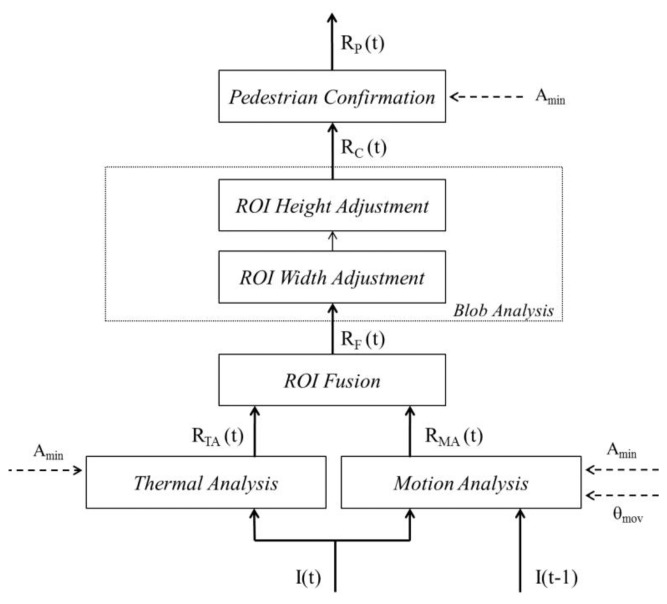
Algorithm for pedestrian ROI extraction in thermal-infrared video.

In parallel, motion information between the current frame *I*(t) and the previous frame *I*(t−1) is performed under *Motion Analysis*. A visual representation of the approach is provided in Figure 1. Notice that the results of *Thermal Analysis* and *Motion Analysis* are fused (*ROI Fusion*) to take advantage of both thermal and motion information provided in the video sequence. *Blob Analysis* validates if a given blob corresponding to a supposed pedestrian contains one or more than one human. Lastly, *Pedestrian Confirmation* validates that a refined blob actually contains a valid pedestrian.

### 2.1. Thermal Analysis

A pedestrian ROI extraction based on thermal information is developed in the thermal-infrared spectrum using the properties already mentioned [15]. Pedestrian candidates are extracted in each image frame, solely based on their thermal properties. A set of restrictions on size and shape are applied on the adjusted candidates to eliminate potential false positives. Each one of the stages is now explained in more detail.

The algorithm starts with the analysis of input image, *I*(t), captured at time t. Image *I* is binarised in accordance with a threshold with the aim of isolating the spots related to the pedestrian candidates. This threshold obtains the image areas containing moderate heat blobs, thus probably belonging to pedestrians (pedestrian candidates). This way, warmer zones of the image are isolated where humans could be present. The threshold *θ_TA_* is calculated in function of the mean (*Ī*) and the standard deviation (*σ_I_*) of image *I*, as shown in Equation (1):

(1) $$\theta_{TA}=\frac{5}{4}(\bar{I}+\sigma_{I})$$

Next, the algorithm performs morphological opening and closing operations to eliminate isolated pixels and to unite areas split during the binarization into mage blobs. A minimum area, *A*_min_–function through triangulation of the distance of the camera to the farthest objective–is established for a blob to be considered to contain one or more humans. The output of *Thermal Analysis* towards *ROI Fusion* is a list of regions of interest (ROIs) denominated *R_TA_*(t).

### 2.2. Motion Analysis

We have previously explained that certain environmental conditions affect negatively the visual contrast in the thermal-infrared spectrum. For example, humans are very hard to find in warm environments where the scene temperature is similar to people's temperature. Yet, if using the motion information in the scene, we can find humans in it since they do not tend to be static during long periods of time. Therefore, *Motion Analysis* is developed to take advantage of the motion information in the scene.

Here, the previous image, *I*(t−1), and the current one, *I*(t), are used. Notice that images are captured a frame rate of 5 images per second, which ensures enough movement and enables processing all the image frames in real-time. An image subtraction and thresholding is performed on these frames. The threshold is experimentally fixed to 16% of the maximum value of a 256 gray levels image; thus, threshold *θ_mov_* takes the value 16. It is calculated that a pixel (*x,y*) is “warm” if:

(2) $$\left|I(x,y,t)-I(x,y,t-1)\right|>\theta_{\text{mov}}$$

Now, ROIs with area superior to *A*_min_ and with a percentage of “warm” pixels greater than a rate threshold (experimentally fixed to 5% of the area of the ROI) are extracted into list *R_MA_*(t).

### 2.3. ROI Fusion

The objective of *ROI Fusion* is to sum up or overlap the ROIs coming from *Thermal Analysis* and *Motion Analysis* to get a unique list of regions of interest *R_F_*(t). We are faced with three possibilities:

(1) A ROI belonging to list *R_TA_*(t) has no common pixel with any ROI belonging to *R_MA_*(t): the ROI from *R_TA_*(t) is included as is in the new list of ROIs called *R_F_*(t).
(2) A ROI belonging to list *R_MA_*(t) has no common pixel with any ROI belonging to *R_TA_*(t): the ROI from *R_MA_*(t) is included as is in the new list of ROIs called *R_F_*(t).
(3) A ROI belonging to list *R_TA_*(t) has some common pixels with a given ROI belonging to *R_MA_*(t): the ROIs from *R_TA_*(t) and *R_MA_*(t) compose a new ROI containing all pixels from the previous ones; this new ROI is included in the new list of ROIs called *R_F_*(t).

Rules (1) and (2) show the possibilities to sum up the ROIs coming from both Thermal Analysis and Motion Analysis. Rule (3) demonstrates the case when both Thermal Analysis and Motion Analysis have detected the same candidates as pedestrians (or at least part of them).

### 2.4. Blob Analysis

This part of the algorithm works with the list *R_F_*(t). This list was obtained at the end of the previous section. At this point, there is a need to validate the content of each ROI to find out if it contains one single human candidate or more than one. Therefore, each detected ROI is individually processed.

#### 2.4.1. ROI Width Adjustment

The first step of *Blob Analysis* consists in scanning *R_F_* by columns, adding the gray level value corresponding to each pixel in that column. This way, a histogram *H*[*i*] is obtained (see Equation (3)), which shows the zones of the current ROI that contain greater heat concentrations:

(3) $$H[i]=\sum_{j}R_{F}(i,j),\forall i$$

A double purpose is pursued when computing the histogram. In first place, we want to increase the certainty of the presence of human heads. Secondly, as a ROI may contain several persons that are close enough to each other, the histogram helps separating human groups (if any) into single humans. This method, when looking for maxima and minima within the histogram allows differentiating among the people actually present in a particular ROI.

So, the histogram *H*[*i*] is scanned to separate grouped humans, if they exist in that ROI. Local maxima and local minima are searched in the histogram to establish the different heat sources with this purpose. To assess whether a histogram column contains a local maximum or minimum, a new threshold is fixed. We are looking for columns where the 60% of their pixels are below the mean gray value of *R_F_*, since those regions are supposed to belong to gaps between two humans. This way the list *R_F_* will form a new list of sub-ROIs *sR_F_*(t). Notice that if each *R_F_* contains a single human, *sR_F_*(t) will be equivalent to *R_F_*(t).

#### 2.4.2. ROI Height Adjustment

All humans contained in a given sub-ROI of list *sR_F_*(t), obtained in the previous section, still possess the same height, namely the height of the original ROI. Now, we want to fit the height of each sub-ROI to the real height of the humans contained in it. For this purpose row adjustment is performed. The calculation is done separately on each sub-ROI to avoid the influence of the rest of image pixels on the result. This threshold uses the value of the sub-ROI mean gray level. Each sub-ROI is binarised in order to delimit its upper and lower limits. After this, a closing operation is performed to unite spots isolated in the binarisation. The newly obtained ROIs are now enlisted into *R_C_*(t).

### 2.5. Pedestrian Confirmation

Now a final stage is needed for each ROI of list *R_C_*(t) to confirm if the human candidate is actually a human. Indeed, some incandescent spots in an image (such as light bulbs or big heat sources in general) can still be confused under certain circumstances with humans due to their heat properties. So an important step consists in verifying if one of these spots is being scanned instead of a human.

For this sake, firstly the human candidate's ROI dimensions are checked. The first check consists in testing the ROI's height/width ratio. If the human candidate's width is larger than its height, the standard deviation of the brightness of the ROI is checked. This is due to the fact that incandescent spots such as lamps or fuses have a low standard deviation since their heat distribution is uniform. On the contrary, humans have different heat concentrations in their body parts, such as the head being warmer than the rest of the body. We have determined experimentally that the standard deviation of the human ROI has to be greater than 12.

The human candidate's area is also required to be above a minimum area *A*_min_ experimentally fixed according to features such as the camera height or the extension of the scenario. Finally, the final list of ROIs containing humans is the output of the people detection algorithm, that is, *R_P_*(t).

## 3. Results and Discussion

### 3.1. Test Environment

**Figure 2.**
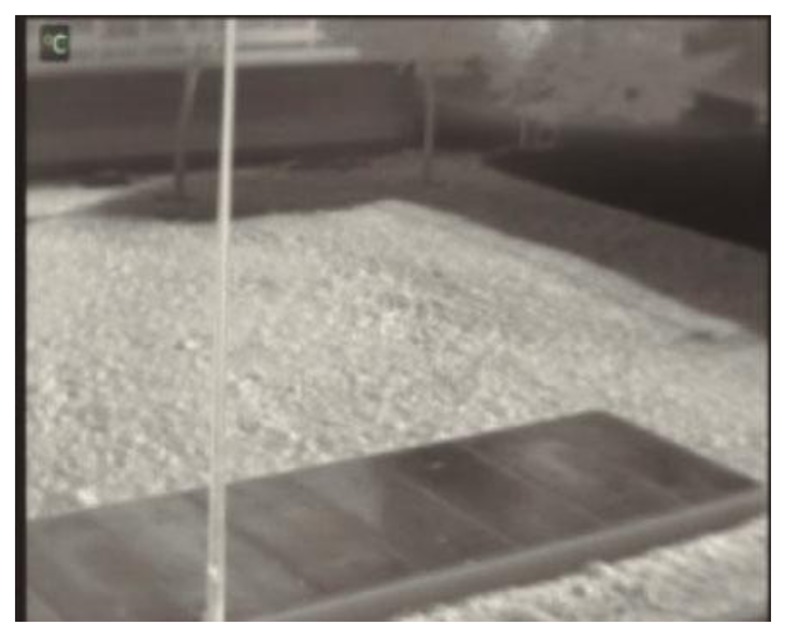
Environment for validating the robustness of the approach.

The selected test environment is an outdoor scenario where a forward-looking infrared FLIR A-320 camera has been placed 6 meters above the ground level. The decision to use an outdoor environment is due to the fact that this kind of scenario offers a greater number of variations in temperature and lighting conditions, whereas an indoor environment is usually more controlled. The scenario does not have any predefined access, so that a pedestrian enters into the scene from the lower limits as well as at the left or right sides of the image. A platform constructed of concrete is located in the lower part of the scene. This material quickly absorbs the temperature of the environment. The same property is also present in the building placed in the scene background. The building shows additional problems for thermal-infrared human detection. The reason is that the thermal-infrared camera automatically performs thermal attenuation, which results in the lack of accuracy in obtaining far objects' temperatures. The attenuation causes the thermal readings of pedestrians to be confused with the temperature of the building, this way hardening their isolation from the scene background. Figure 2 shows an image of the scenario as captured by the FLIR camera.

### 3.2. Test Sequences

To evaluate our algorithms, we have tested a number of sequences at different temperatures and under different conditions. The main objective is to cover the maximum possible number of situations, both in complexity and variation of temperature. To do this, it was decided to include a range of winter and summer temperatures, ranging between −2° and 33°. We have also sought to work under different weather conditions from snow to sunshine. In addition, we used situations of varying complexity, from a single human walking on the scene up to three people meeting, with various actions that pedestrians can perform on an exterior scene. These actions range from attitudes in which humans are easy to detect such as walking or running to other more difficult, because people change the proportions the space they occupy, such as bending, sitting or even lying on the floor. Next, the different recorded sequences are described. Each of these twelve sequences is referred to by the temperature at which it was captured, followed by the atmospheric conditions at the time of the recording.

**Figure 3.**
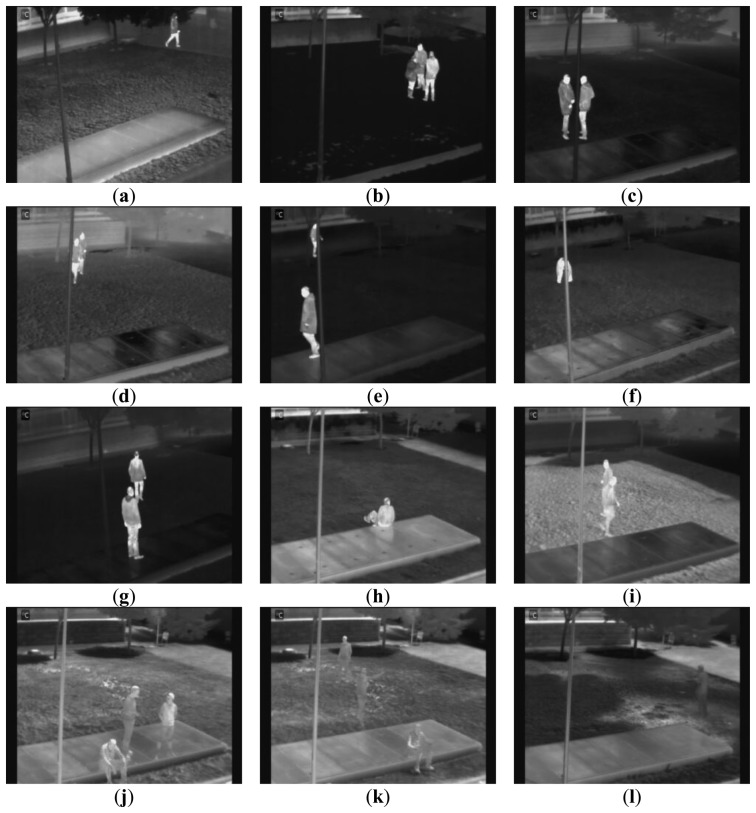
Example frames of the twelve sequences. (**a**) −2°*Foggy*; (**b**) 2°*Snowy*; (**c**) 3°*Sunny*; (**d**) 8°*Night*; (**e**) 9°*Cloudy*; (**f**) 10°*Cloudy*; (**g**) 15°*Dawning*; (**h**) 15°*Cloudy*; (**i**) 18°*Sunny*; (**j**) 23°*Sunny*; (**k**) 28°*Sunny*; (**l**) 33°*Sunny*.

- Sequence −2°*Foggy* features a human in the scenario (see Figure 3a). The pedestrian is mostly walking, but also performs actions such as crouching, running or sitting in the central concrete platform. The sequence was recorded in a moment where fog was partially covering the scene. It is not difficult to distinguish humans in the thermal-infrared spectrum, except when they are approach the building.
- Sequence 2°*Snowy* was recorded after a snowfall, and therefore all the ground appears covered by snow (see Figure 3b). Behaviors within the sequence have a high complexity. During the course of the sequence three human repeatedly appear together (so that the algorithm has difficulty to separate them, as they often occlude each other). Various activities such as running, walking, bending or dropping items on the floor are made.
- Sequence 3°*Sunny* (see Figure 3c) starts with a human walking in the environment. Sometimes, he/she carries out different actions such as crouching. Later, a second human is walking in different trajectories. Finally, both humans cross their paths, meeting on the concrete platform.
- Another sequence named 8°*Night* was recorded to evaluate the performance of the approach under night conditions (see Figure 3d). The thermal-infrared spectrum introduces a number of problems. Indeed, buildings in the environment are still warm due to the heat accumulated during the day hours. Thus, the buildings are sometimes confused with humans walking in front of them. The sequence features two people walking in the scenario, occasionally crossing their paths.
- Sequence 9°*Cloudy* was captured on a cloudy day (see Figure 3e), and in it, two people follow random paths across the stage. In the thermal-infrared spectrum humans remain easily distinguishable from the rest of the environment.
- Now, sequence 10°*Cloudy* presents a simpler version of the above sequence, with one person walking across the stage and performing various actions such as bending and strolling along the worst lit areas of the stage, as are the shadows of the trees (see Figure 3f).
- Sequence 15°*Dawning* was filmed at sunrise (see Figure 3g). During the scene, gradual changes in illumination and temperature are recorded, starting with the very dim lighting and increasing as the sequence advances. In the sequence two pedestrians continuously gather and meet, so that there are many occlusions.
- In the sequence 15°*Cloudy* some more complex actions are performed by a single human, such as sitting in the central platform (see Figure 3h). The temperature rise causes the apparition of human reflections on the concrete platform, this way augmenting the difficulty for human detection in the infrared spectrum.
- Sequence 18°*Sunny* contains groups of pedestrians (see Figure 3i). There is also the added difficulty that at this temperature the heat of the lawn and the environment in general increases, making it harder to distinguish humans, even to the naked eye in the captured frames in the thermal-infrared.
- Sequence 23°*Sunny* (see Figure 3j) is much more complex than before, because, this time increases to three the number of humans who walk through the scene and gather several times, sitting or simply crossing. Again, the high temperature makes it difficult to distinguish humans in the thermal-infrared spectrum, the area above the concrete platform being especially critical.
- Sequence 28°*Sunny* augments the difficulty of thermal-infrared pedestrian detection with the apparition of up to three pedestrians walking in the scene and performing actions such as sitting, crossing their paths, and meeting. The high temperature makes it quite difficult to distinguish humans in the infrared spectrum, especially on the concrete platform (see Figure 3k).
- Finally, sequence 33°*Sunny* was recorded with much heat. Humans are almost indistinguishable from the background in the thermal-infrared spectrum and appear always cooler than the rest of the environment (see Figure 3l).

### 3.3. Assessment Criteria

Some measures widely used by the computer vision community, such as recall, precision and F-score, were considered to evaluate the performance of the previously described segmentation algorithms. These measures are calculated as shown in Equations (4)–(6), respectively:

(4) $$\text{recall}=\overset{TP}{\;}/\underset{TP+FN}{\;}$$

(5) $$\text{precision}=\overset{TP}{\;}/\underset{TP+FP}{\;}$$

(6) $$F-\text{score}=\overset{\text{recall}}{\;}/\underset{\text{precision}+\text{recall}}{\;}$$

where TP (true positives) is the amount of correct detections in the sequence, FP (false positives) are the mistaken detections gotten and FN (false negatives) is the amount of humans really present in the scene but not detected.

The precision shows the percentage of true positives with respect to the total number of detections, *i.e.*, the probability of detections which really correspond to a human. On the other hand, the recall shows the probability of a human on the scene to be really detected. Finally, F-score is a weighted average, which provides an overall vision of the system performance, considering precision and recall.

### 3.4. ROI Extraction Results

The results obtained are shown in Table 1. The first conclusion to be drawn is quite obvious. In general, the thermal-infrared spectrum is suitable for detecting human under low and medium recorded temperatures. Notice that the sequence captured at 8° shows worse results, as was recorded in the early hours of the night and the temperature had not yet fallen. Under all these thermal conditions the *F-score* is maintained over a good 0.83 value.

However, the performance declines drastically when the temperature of the scene rises above 20°. This is due to the fact that the thermal radiation of humans is very similar to the temperature of the buildings. Indeed, the sun warms the scene directly, affecting the elements of it. This has a significant impact on the final sequence, in which humans are totally “unified” with the environment and the distinction is almost impossible, even for a human observer who is supervising the frames captured in thermal-infrared. Notice that the *recall* value falls down dramatically by only incrementing a few degrees in the ambient temperature. The 33°*Sunny* sequence shows a very bad performance (0.03).

Some other conclusions can also be drawn. These are related with atmospheric environmental conditions. In accordance with the results obtained in Table 1, we can conclude that there is no difference between snowy, cloudy and sunny conditions beneath a given temperature (around 20°). Indeed, the *recall* and the *F-score* are always kept above excellent 0.91 and 0.94 values, respectively. However, notice that the foggy sequence drops the value of *recall* down to 0.71, which is still a good value, but nor comparable to other scores obtained for a similar temperature.

This way we can conclude that pedestrian ROI extraction in the thermal-infrared spectrum provides excellent results for low and medium ambient temperatures, but the results could be affected by some specific weather conditions.

## 4. Conclusions

This article has provided comprehensive information about tests that have been conducted to evaluate the performance of a new algorithm developed for detecting human in thermal-infrared video. The paper has described our thermal-infrared pedestrian ROI extraction algorithm. Then, the evaluation of the proposal has been introduced in detail. The results allowed us to assess the validity of our thermal-infrared proposal to robustly detect pedestrians under varying dynamic outdoor conditions. We have also been able to study under which weather conditions and temperatures the approach is consistent and throws from good up to excellent detection results for videos captured by a forward-looking infrared FLIR A-320 camera.

## Figures and Tables

**Table 1.** Results of pedestrian ROI extraction in thermal-infrared.

**Sequence**	**TP**	**FP**	**FN**	***Recall***	***Precision***	***F-Score***
2°*Foggy*	11,928	147	4,784	0.71	0.99	0.83
2°*Snowy*	3,224	163	156	0.95	0.95	0.95
3°*Sunny*	2,902	295	44	0.98	0.91	0.94
8°*Noche*	4,787	766	1,112	0.81	0.86	0.83
9°*Cloudy*	1,618	61	105	0.94	0.96	0.95
10°*Cloudy*	1,827	12	22	0.99	0.99	0.99
15°*Dawning*	3,957	12	293	0.93	1.00	0.96
15°*Cloudy*	1,684	51	160	0.91	0.97	0.94
18°*Sunny*	2,185	19	176	0.93	0.99	0.96
23°*Sunny*	2,174	363	1,448	0.60	0.86	0.71
28°*Sunny*	3,077	160	4,861	0.39	0.96	0.55
33°*Sunny*	123	23	3,393	0.03	0.84	0.04
